# A mechanically validated open-source silicone model for the training of gastric perforation sewing

**DOI:** 10.1186/s12909-023-04174-8

**Published:** 2023-04-19

**Authors:** Lukas Warnung, Stefan Sattler, Elmar Haiden, Sophie Schober, Dieter Pahr, Andreas Reisinger

**Affiliations:** 1 Department of Anatomy and Biomechanics, Division Biomechanics, Karl Landsteiner University of Health Sciences, Dr. Karl-Dorrek-Straße 30, Krems, 3500 Austria; 2 Department of Surgery, University Hospital Tulln, Alter Ziegelweg 10, Tulln, 3430 Austria; 3 Medical Science and Human Medicine study programme, Karl Landsteiner University of Health Sciences, Dr. Karl-Dorrek-Straße 30, Krems, 3500 Austria; 4 Institute for Lightweight Design and Structural Biomechanics, University of Technology Vienna, Getreidemarkt 9, Wien, 1060 Austria; 5 Division of Radiotherapy-Radiation Oncology, University Hospital Krems, Mitterweg 10, Krems, 3500 Austria; 6 Karl Landsteiner University of Health Sciences, Dr. Karl-Dorrek-Straße 30, Krems, 3500 Austria

**Keywords:** Gastric perforation, Silicone model, Surgical training, Needle penetration, Haptic evaluation

## Abstract

**Background:**

Gastrointestinal perforation is commonly seen in emergency departments. The perforation of the stomach is an emergency situation that requires immediate surgical treatment. The necessary surgical skills require regular practical training. Owing to patient`s safety, in vivo training opportunities in medicine are restricted. Animal tissue especially porcine tissue, is commonly used for surgical training. Due to its limiting factors, artificial training models are often to be preferred. Many artificial models are on the market but to our knowledge, none that mimic the haptic- and sewing properties of a stomach wall at the same time. In this study, an open source silicone model of a gastric perforation for training of gastric sewing was developed that attempts to provide realistic haptic- and sewing behaviour.

**Methods:**

To simulate the layered structure of the human stomach, different silicone materials were used to produce three different model layups. The production process was kept as simple as possible to make it easily reproducible. A needle penetration setup as well as a systematic haptic evaluation were developed to compare these silicone models to a real porcine stomach in order to identify the most realistic model.

**Results:**

A silicone model consisting of three layers was identified as being the most promising and was tested by clinical surgeons.

**Conclusions:**

The presented model simulates the sewing characteristics of a human stomach wall, is easily reproducible at low-costs and can be used for practicing gastric suturing techniques.

**Trial registrations:**

Not applicable.

**Supplementary Information:**

The online version contains supplementary material available at 10.1186/s12909-023-04174-8.

## Background

The human stomach, consists of four different layers: the mucosa, the submucosa, the muscularis externa and the serosa. Three different layers form the mucosa: the surface epithelium (containing gastric pits and gastric glands), the lamina propria, and the muscularis mucosae. The muscularis ext. consists of a longitudinal external muscle layer, a circular middle muscle layer and an oblique internal muscle layer [1, 2].

Different pathologies (gastric ulcer, inflammation, malignancies, trauma) can lead to gastric perforation. This indication can lead to a life-threatening disease and therefore requires a quick and efficient treatment.

**Fig. 1:**
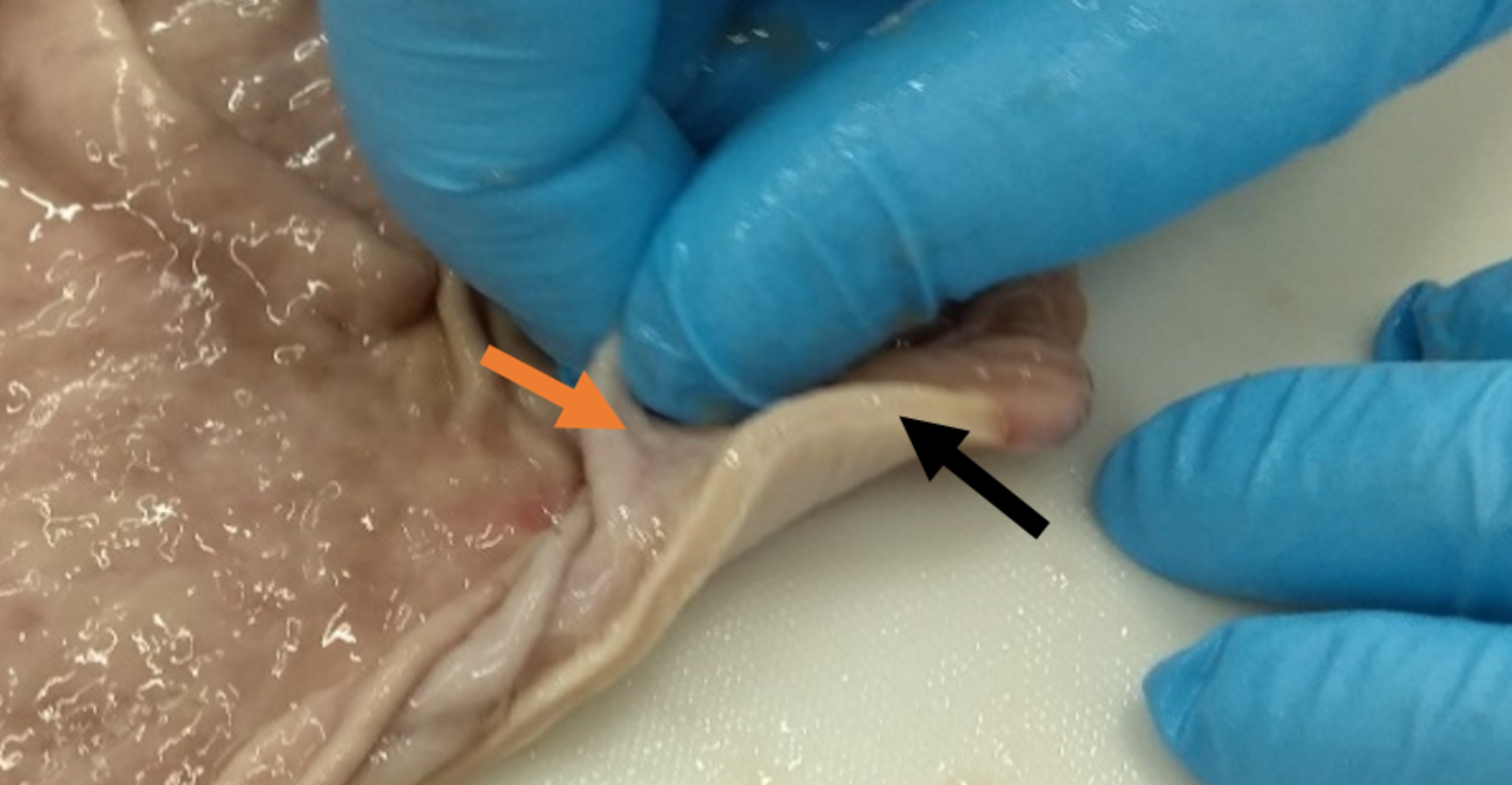
Differentiation of non-sewing and sewing layer of a porcine stomach wall. As shown, the muscularis ext., covered with serosa (= layer that is sewed; black arrow) can be distinguished from the mucosa (layer not sewed; orange arrow)

The treatment of a stomach perforation is done either by an open or laparoscopic surgery whereat the muscularis externa is closed by sewing and the mucosa remains untouched (Fig. 1) [3].

Primary closure by interrupted sutures, closure by interrupted sutures covered with a pedicled omentum on top of the repair (Cellan-Jones repair) and plugging the perforation with a free omental plug (Graham patch) are the most common techniques [4].

Those surgical techniques require much training and patient`s safety during surgeries is highly dependent on the surgeons skills [5, 6] showed that even a one-day surgical-skill training course for medical students improved their surgical skills. Moreover, simulation-based training is beneficial in, for instance training laparoscopic surgery [7].

However, surgical training in humans has a number of restrictions such as patient safety issues, ethical and economic considerations or lack of exposure to specific surgical procedures [8–12].

Instead, animal tissue is often used in research and education, especially if there are no sufficient non-cadaveric training models on the market. So far, surgeons are still using porcine stomachs for training gastric perforation surgery. The digestive system and, the structure of the stomach wall of pigs is similar to all other monogastric mammals. Therefore, the stomach of a pig has a similar structure and function as a human stomach. Additionally, porcine stomachs are usually easily available and of low-costs [13].

The use of animal tissue has also many disadvantages and limitations, for example the short time frame where the tissue is usable and the need for the required infrastructure (cooling capabilities, wet areas), [14–19]. Furthermore, ethical aspects are still a compelling reason for using artificial training models instead of biological organs or tissue [20, 21].

Therefore, artificial models similar to biological tissue are desirable substitutes for real biological tissues and organs for applications in research, medical training, and teaching. However, accurately mimicking the mechanical and haptic properties of a tissue or an organ is a challenge. In order to mimic soft tissue, potential materials include soft compliant substances such as silicones, gelatine, or hydrogels [22].

Many different silicones are available on the market, providing a variety of flexibility and strength. To combine the advantages of a long lifetime, realistic mechanical properties, similar haptic conditions and a good working environment, a silicone training model could be a good surrogate [23]. It was already shown in [24] and [25] that silicone models cast in 3d printed moulds improve the skill level of surgeons when used in surgical training.

Within this study, a set of artificial silicone models of a stomach wall with realistic mechanical and haptic properties is developed to be used in training of gastric surgery. To support the open-source notion, the models should be easy to manufacture and cheap to copy.

The silicone models are compared with fresh porcine stomach wall in terms of needle penetration force characteristics in a mechanical test setup. Additionally, a systematic haptic comparison, including appearance and piercing- and tear out forces was performed. Similar investigations for testing artificial tissue have already been able to achieve some results, such as in [26–28].

## Methods

### Silicone model design

After many pre-tests, three different silicone model layups with similar properties were identified as meaningful imitations of a real stomach wall. The models were produced from silicone types of the brand Eco-Flex (00–10, 00–20, 00–30) and Mold Star™ 30/1 Shore 30 A (KauPo Plankenhorn e.K., Spaichingen, Germany) and open cell PU foam sheet with 5 mm thickness and an area density of 96 g/m², bought from a hardware store.

**Fig. 2:**
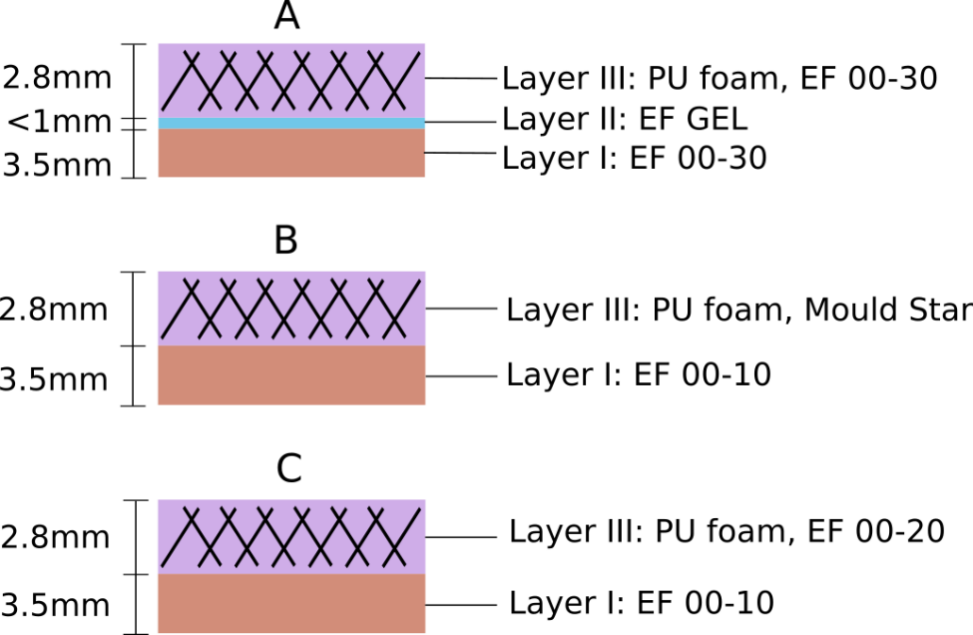
Structure of the developed silicone models A, B and C. Note that only model A contains an additional layer (layer II) as representation of the submucosa and muscularis mucosa

The anatomical layers of the stomach wall were modelled by representative silicone layers (Fig. 2). From bottom to top:

- Silicone layer I represents the soft and wrinkled mucosa and is thus designed to be very soft.
- Silicone layer II, present in model A only, aims to mimic the submucosa and muscularis mucosa as a soft and thin middle layer that allows for some transverse sliding.
- Silicone layer III forms the muscularis externa and the serosa, which are firm and stiffer tissues. As in the real gastric perforation surgery, only this layer is sewed. (sewing layer)

Based on the requirement to mimic the haptic properties and sewing performance of a real stomach, the constituents of the silicone layers are chosen to represent the mechanical properties of the anatomical layers as close as possible. In particular, the sewing layer needed to be reinforced with an underlying fibrous structure to establish a realistic sewing behaviour with less tear out. Therefore, in all three silicone models, PU foam was introduced to layer III.

**Fig. 3:**
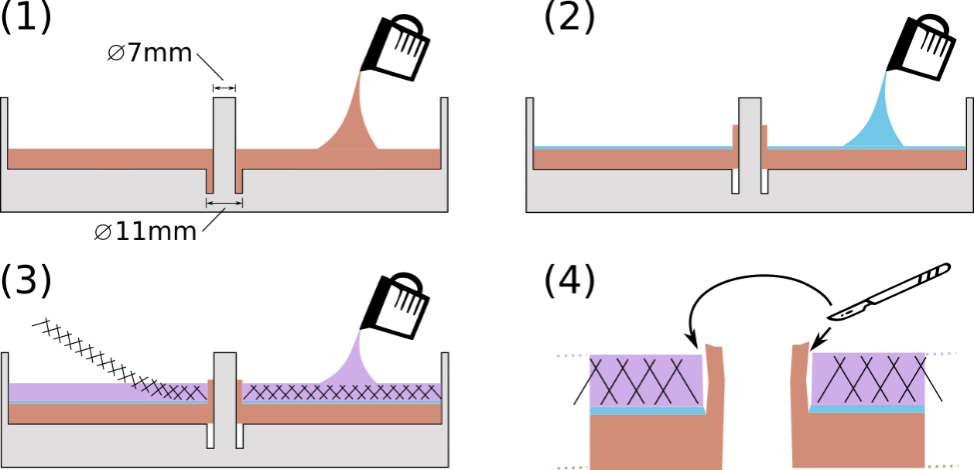
Schematic fabrication process: Step (1): Casting of the first layer after lubrication the cast with oil. Step (2): Flipping the first layer within the mold and casting of layer II (model A only). Step (3): Introducing the foam and casting layer III. The PU foam can also be prepared separately (soaked in silicone) and placed on the previous layer. Step (4): after hardening, the model is removed from the mold. The protrusions were separated with a scalpel from the sidewalls of layer II and III

The perforation of the stomach wall is modelled as a hole with 7 mm in diameter through all layers (Fig. 3). To copy a potential protrusion of the mucosa and to add the surgical challenge of pushing it back while sewing, layer I is raised like a collar through the perforation hole. The collar diameter is 11 mm.

Layer upon layer were cast in one mold. To decrease the curing time of the first layer to 15 min, a mild heat of 55 °C was applied by putting the mold on the head bed of a FDM 3D-printer. However, the used silicones would also cure at room temperature, but slower. By flipping layer I, the protrusion of the mucosa is raised. Then, an intermediate layer II was added to model A. After that, the silicone for the third layer was applied directly upon the previous layer, including the silicone soaked PU foam sheet to add additional strength [23, 29]. After curing all layers and removing the model from the mold, the protrusion was loosened from layers II and III by a circular cut with a scalpel. This enables the protrusion to be pushed in later on in the simulated surgery as additional surgical challenge. With this technique, three different silicone models were created (sample number of n = 5 per model).

**Fig. 4:**
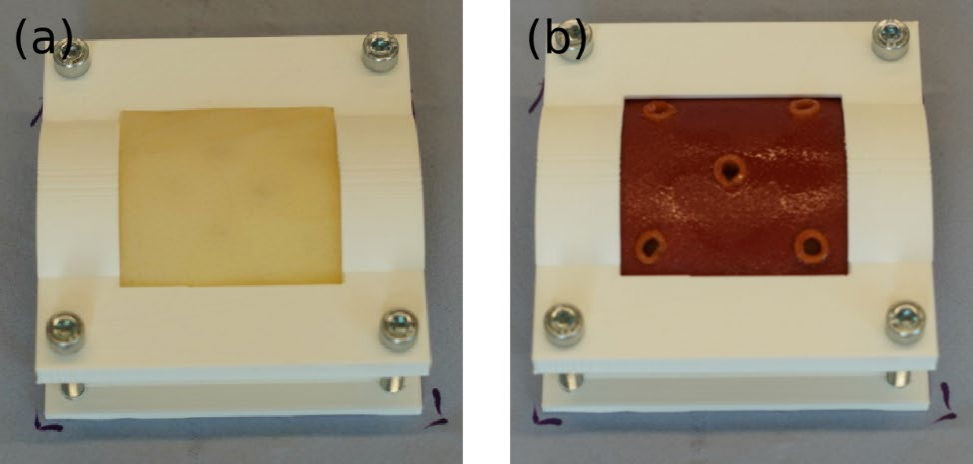
(a) 3D-printed test stand with a foam padding serving as an underlayer for the silicone model to provide more flexibility. (b) finalised silicone training model of a gastric perforation: The model is fixed in the device, muscle side (sewing area) with 5 perforation holes and protruding mucosa (non-sewing area)

Furthermore, a 3D printed test stand (Fig. 4) is added, to render the sewing situation to a realistic level. The silicone model is supported via a foam padding (Fig. 4a), to create a convex surface that would move realistically upon touch, such as the real organ would do, which is one of the challenges of a stomach perforation surgery.

### Preparation of porcine stomach samples

As the aim of this study is the design of realistic silicone models, they need to be systematically compared to real stomach wall tissue samples.

**Fig. 5:**
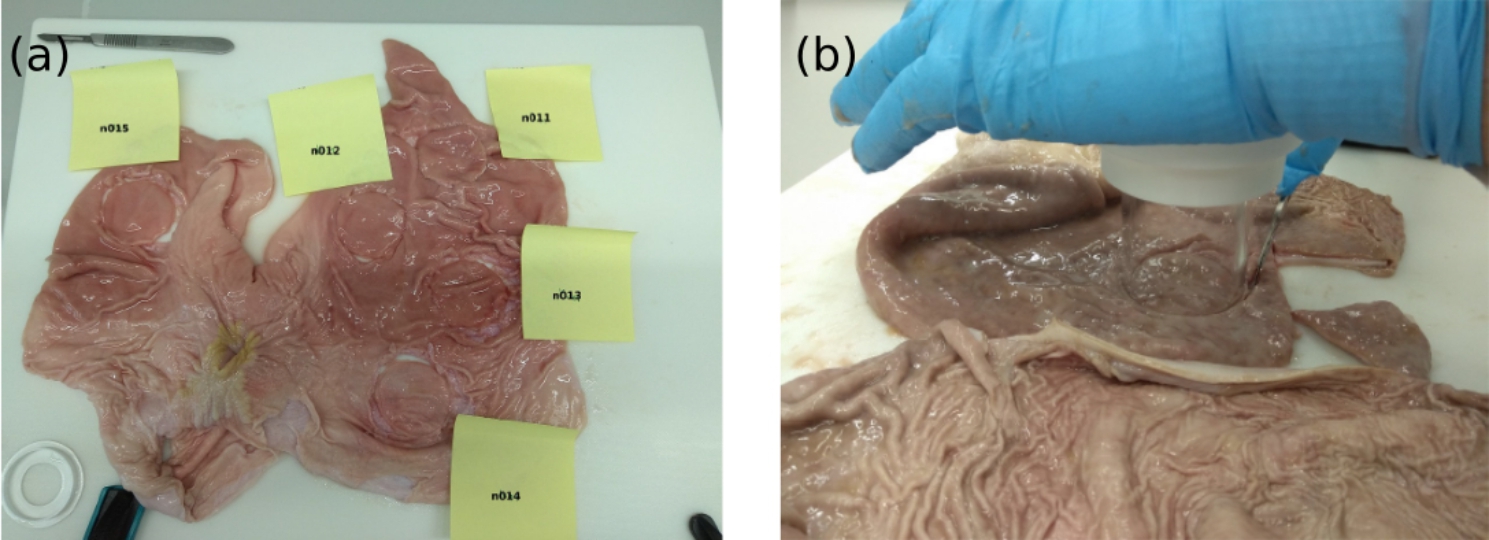
(a) Circular samples cut out from porcine stomach. (b) Cutting was done with a scalpel using the test cylinder as a template

For the use as reference tissue, five pig stomachs were obtained from a butcher. The stomachs were transported to the laboratory in a vacuum bag and stored ~ 48 h at 4 °C until further processing. Five circular samples per pig stomach were prepared, giving a sample number of n = 25 (Fig. 5a). To cut out the disk-shaped samples with a diameter of 48 mm, the cylinder of the test set up served as a template (Fig. 5b). The circular shape allows for mounting the samples in the needle penetration test setup that is described below. In the same way, n = 5 disk shaped samples were cut from each of the silicone model types.

### Haptic testing

**Fig. 6:**
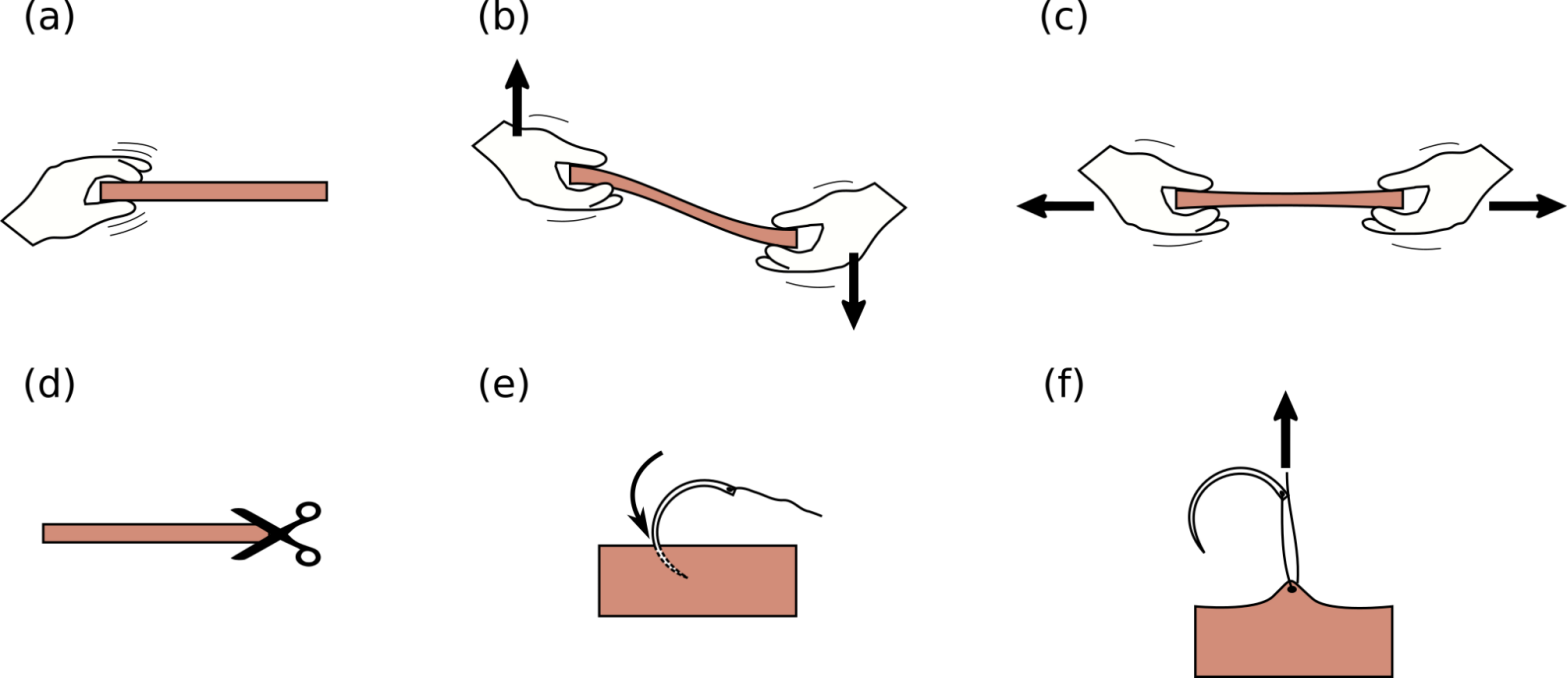
Haptic evaluation aspects. (a) Overall haptics evaluated by touching, (b) out of plane stiffness evaluated by transverse deformation, (c) in-plane stiffness evaluated by stretching, (d) cutting resistance evaluated by cutting with scissors, (e) piercing resistance evaluated by penetrating with a needle, (f) tear resistance evaluated by pulling a thread

Haptic properties were evaluated on fresh porcine stomach and the three different silicone models in a comparative ranking system. Therefore, clearly defined qualities were evaluated (Table 1; Fig. 6). These defined qualities were overall feeling to touch of the model, stiffness in transversal direction, stiffness in longitudinal direction, subjective force needed to cut with a scalpel, subjective force needed to penetrate with a needle and resistance of the sample against tear out of the thread. Those parameters were ranked subjectively from 1 to 4 by a selected group of surgeons and technicians according to Table 1.

**Table 1:** Overview and evaluation of the haptic parameters of the silicone models and the porcine stomach wall

Haptic parameter	Rank 1	Rank 4
Overall structure (Fig. 5a)	Most similar to porcine sample	Least similar to porcine sample
Stiffness in transverse direction (flexibility) (Fig. 5b)	Easiest to move in transversal direction	Hardest to move in transversal direction
Stiffness in longitudinal direction (Fig. 5c)	Elongates easiest	Most resistant to elongation
Subjective force needed to cut (Fig. 5d)	Easiest to cut	Hardest to cut
Subjective force needed to penetrate (Fig. 5e)	Easiest to penetrate	Hardest to penetrate
Durability against tearing of the thread (Fig. 5f)	Sample which is torn the easiest by the thread	Sample which is torn the least by the thread

### Mechanical needle penetration test

Aside from general haptic properties, the sewing characteristics of the artificial model should be as close as possible to a biological stomach wall. Therefore, a mechanical test set up was designed that allows to penetrate artificial and biological tissues with a needle in a controlled manner while measuring force and displacement.

**Fig. 7:**
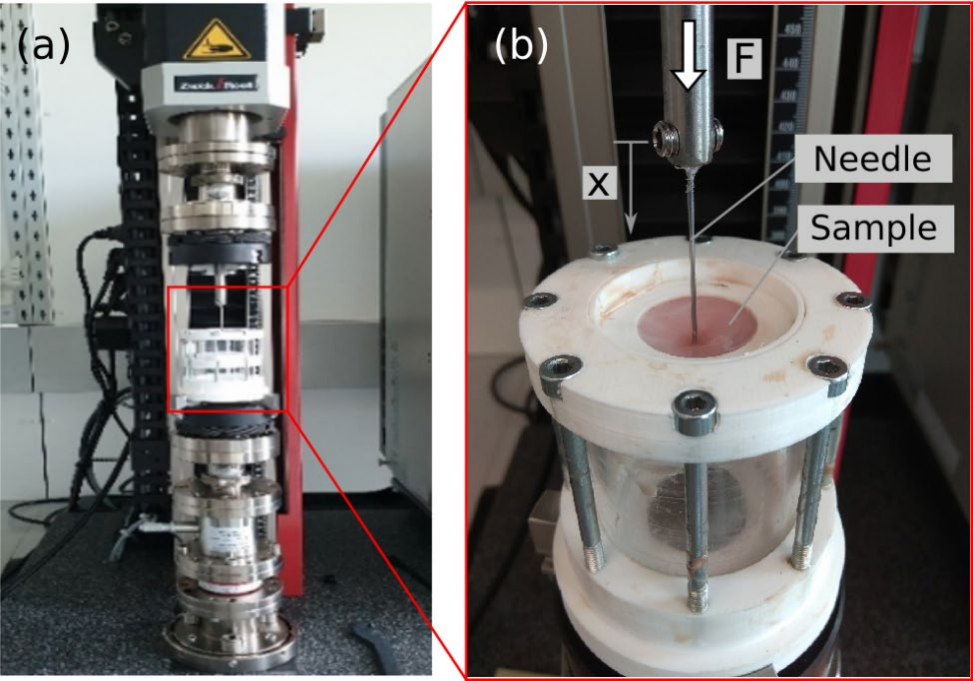
(a) axial testing machine with mounted test setup. (b) needle penetrating the sample while force (F) and displacement (x) is recorded

The test set up consists of a 3d printed sample holder (Fig. 7), in which the sample disk is mounted and a moveable needle to penetrate the sample. The transparent cylinder is made of acrylic glass. The porcine samples were mounted so that the mucosa tissue is facing upwards. Eight M6 screws were used to hold the separate parts together. For penetrating the specimen, a needle (Ethicon TMII, Polyamide 6, 2 − 0 (3 PH. Eur.), EH7625, STAW 65 mm) in combination with a stainless steel rod is mounted to the testing machine (zwickiLine 2,5kN, Zwick Roell GmbH, Ulm, Germany). The standard 2,5 kN load cell and the machine displacement were used as output signals. The loading protocol was: ramp loading with 3 mm/min, travel length of 30 mm and immediate unloading with the same speed. The test was started at the position of the first contact between needle and tissue and from there the displacement x was measured.

### Statistical test

To compare the mechanical test results between the porcine sample group and the three different silicone sample groups, a standard two-sample t-test assuming different variances was used.

## Results

### Haptic test

The porcine samples and silicone samples were tested and ranked after the same tests: touching and deforming the tissue, force needed to cut, needle piercing and thread tear out (See also Fig. 6). The following subjective perceptions were made:

#### Porcine sample

Both surfaces of the porcine samples were sticky. The mucosa is very soft while the muscularis is stiffer and more structured. After pressing or stretching the tissue, it goes back to its original state and can therefore be regarded as elastic. The piercing process is characterized by the relative high initial forces, to break through. Due to the slippery environment, the thread easily slides through the pierced skin layers. The tissue resisted against the tear out attempt.

#### Silicone samples

All silicone models are robust against mechanical deformation. When initiating the needle penetration, the silicone sample accepted the piercing more easily than the porcine sample. However, because the silicone tissue is not slippery, higher forces must be applied on the suture material to gliding through the tissue.

When ranking the haptic impressions gathered from porcine stomach and silicone models, silicone model A is the most similar model compared to porcine stomach considering haptic parameters, as it is the closest in four haptic attributes (Table 2).

**Table 2:** The ranking of the haptic parameters was done under subjective conditions rated by a selected group of surgeons and technicians: porcine stomach serves as reference. The most similar model for the respective parameter is marked with a *. Altogether, silicone model A is the most similar model to porcine stomach regarding haptic parameters

	Overall structure (Fig. 5a)	Stiffness in transverse direction (flexibility) (Fig. 5b)	Stiffness in longitudinal direction (Fig. 5c)	Subjective force needed to cut (Fig. 5d)	Subjective force needed to penetrate (Fig. 5e)	Durability against tearing of the thread (Fig. 5f)
**Porcine stomach** ***(reference)***	1	1	4	2	2	4
**Silicone model A**	2*	3	2	3*	3*	3*
**Silicone model B**	4	4	3*	4	4	1
**Silicone model C**	3	2*	1	1*	1*	2

### Mechanical needle penetration test

**Fig. 8:**
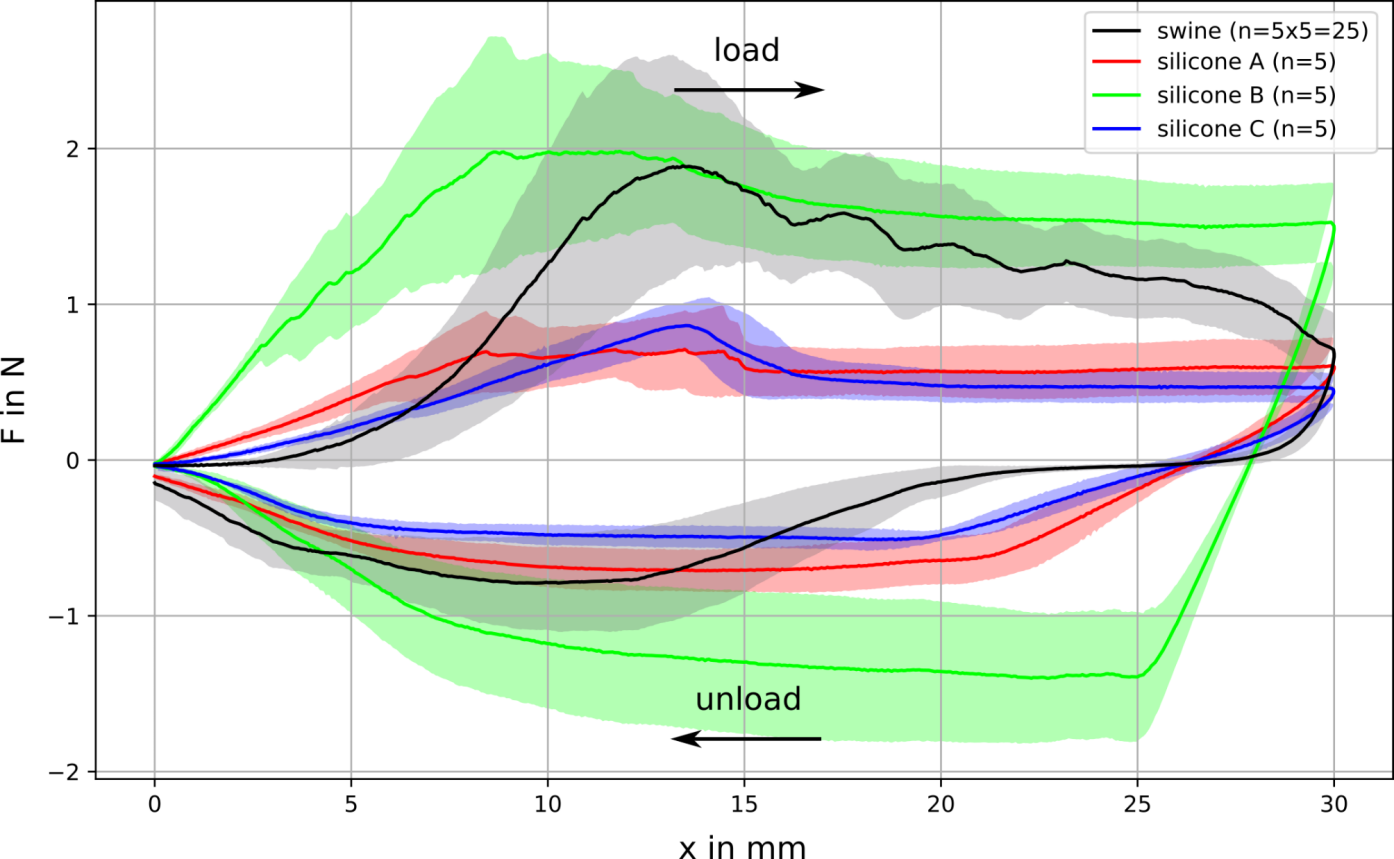
Average force—displacement data of the needle penetration tests of the porcine samples and the silicone models A, B and C. The solid black line represents the averaged data across a sample group including the standard deviation (grey area)

Five samples of each of the three silicone models and five samples of each of the five porcine stomachs were tested in the needle penetration setup and force vs. displacement data were recorded (Fig. 8).

The porcine stomach wall exhibits a “toe”-region at the beginning of the test, indicated by the slow nonlinear increase in force in the first 5 mm. The standard deviation of the porcine data was high, compared to silicone model A and C. Model B has a comparably high standard deviation, and even higher in the unloading part of the curve. Silicone model B showed the best agreement with the porcine samples in the loading phase. Which is underpinned in the maximum force measurement, where model B reaches F_max._ = 2.269 N ± 0.76 N and the comparable porcine samples shows F_max._ = 2.297 N ± 0.498 N. The maximum force for model A (F_max._ = 0.796 N ± 0.252 N) and model C (F_max._ = 0.907 N ± 0.189 N) is not comparable to the maximum force for porcine samples. In the unloading part, models A and C had similar low force response as the biological samples, as verified by the force measurements in the maximum travel region of 30 mm. The silicone model A (F_30mm_ = 0.591 N ± 0.198 N) and C (F_30mm_ = 0,441 N ± 0.114 N) show a comparable force measurement in compare to the porcine sample (F_30mm_ = 0.673 N ± 0.227 N). Model B shows still a force value of F_30mm_ = 1.483 N ± 0.280 N.

### Statistical evaluation of force measurement

**Fig. 9:**
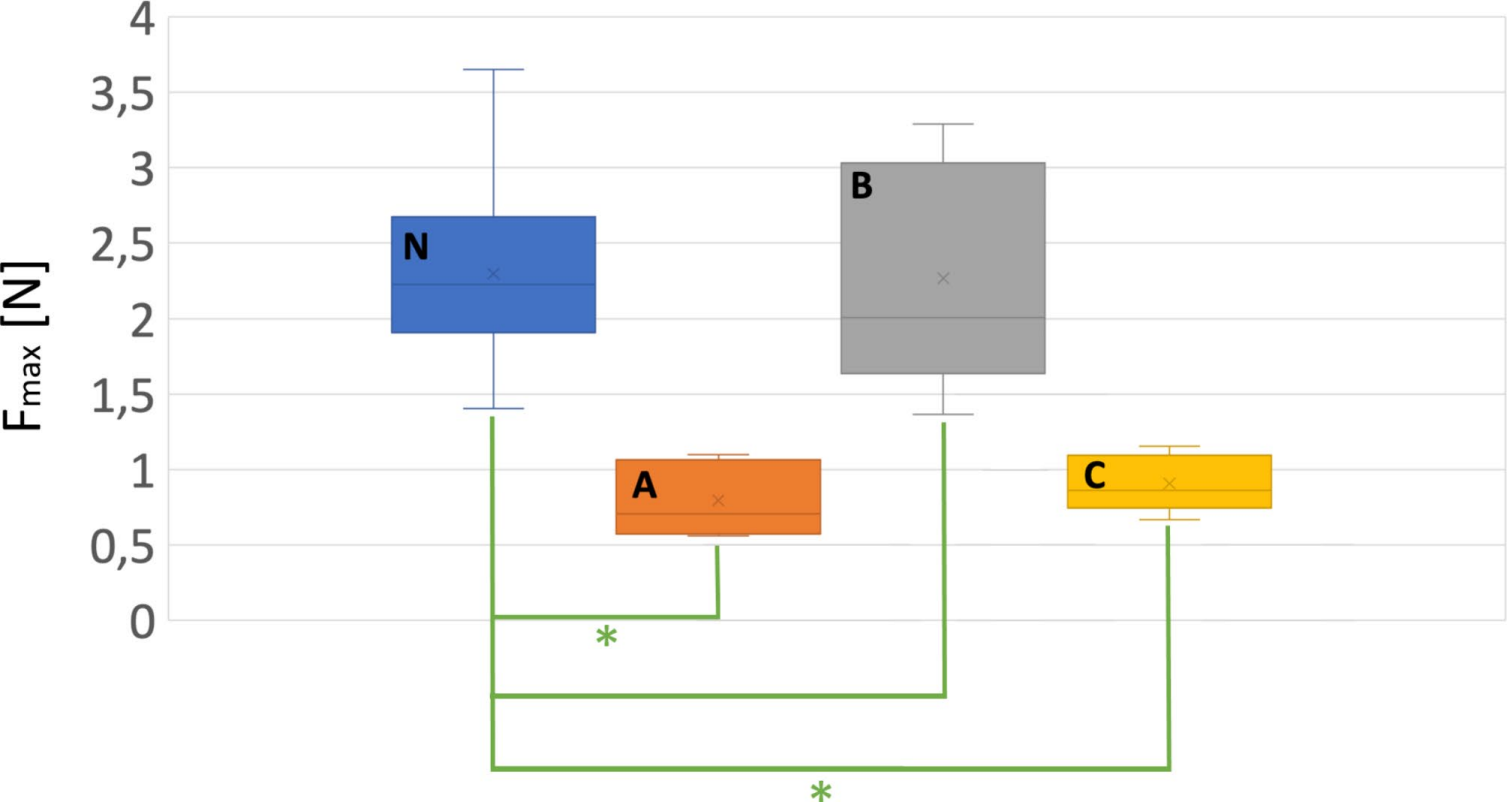
T-test with respect to the maximum force of the porcine samples (N) and the respective silicone models (A,B,C). The asterisk symbol (*) indicates a significant difference considering an alpha level of α = 0.05 (P-values: P_N−A_ = 3.62E-07, P_N−B_ = 0.94, P_N−C_ = 6.13E-09)

**Fig. 10:**
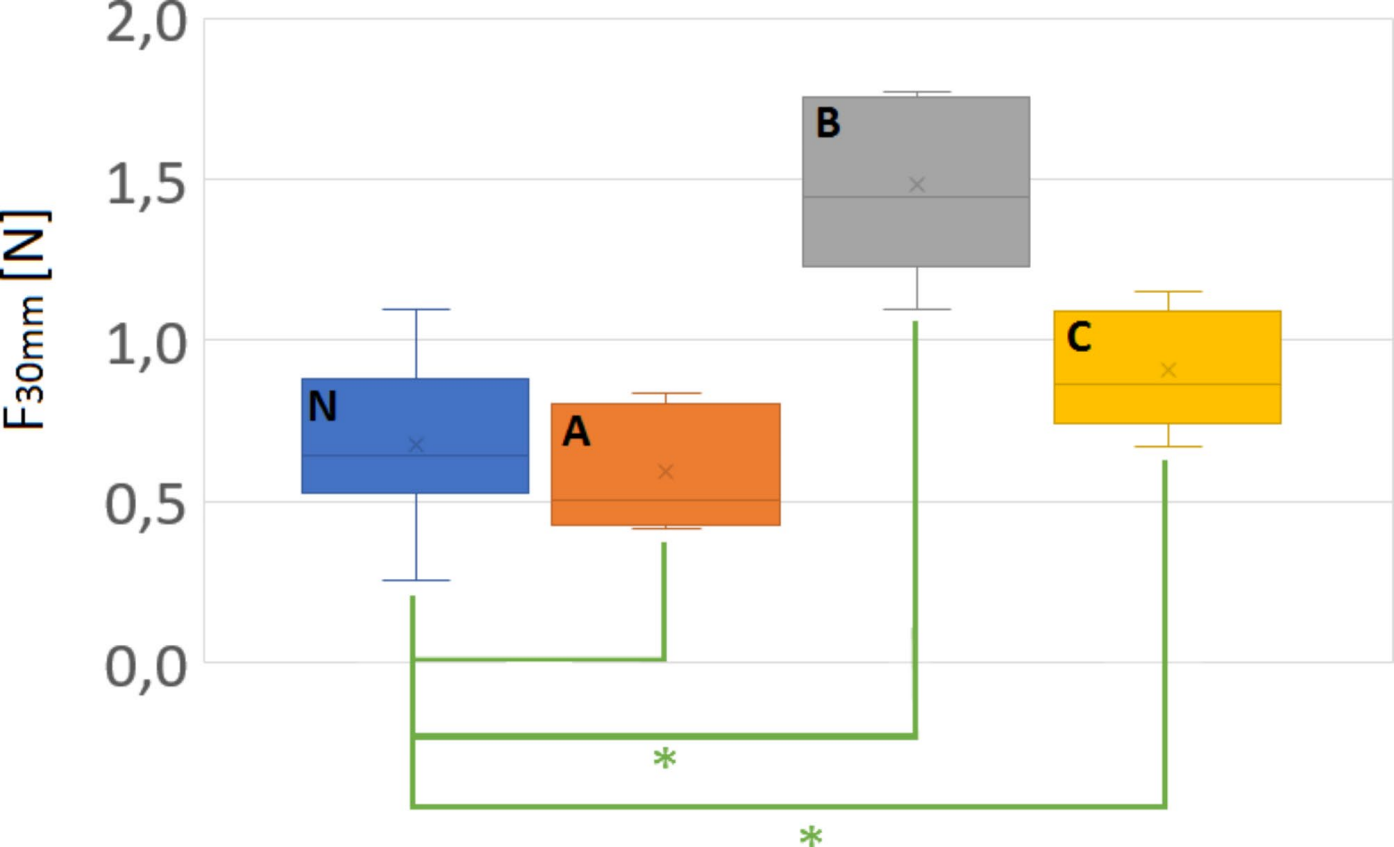
T-test with respect to the force at the maximum travel of 30 mm. The porcine samples (N) and the respective silicone models (A,B,C). The asterisk symbol (*) indicates a significant difference considering an alpha level of α = 0.05 (P-values: P_N−A_ = 0.44, P_N−B_ = 0.16E-02, P_N−C_ = 0.55E-02)

To compare the three silicone samples A, B and C to the porcine samples N in a quantitative way, a statistical analysis was done. Hereby a t-test was performed between the silicone models and the porcine samples, respectively. The significance level of α = 0.05 was used. Results show that only model B did not have a significantly different maximum force (Fig. 9) compared to the porcine samples N and can be therefore regarded as the most realistic model. On the other hand, in the unloading part at the maximum travel of 30 mm, only model A showed no significantly different force (Fig. 10), indicating that model A is more similar to the porcine model.

### The application: a simulated surgery

**Fig. 11:**
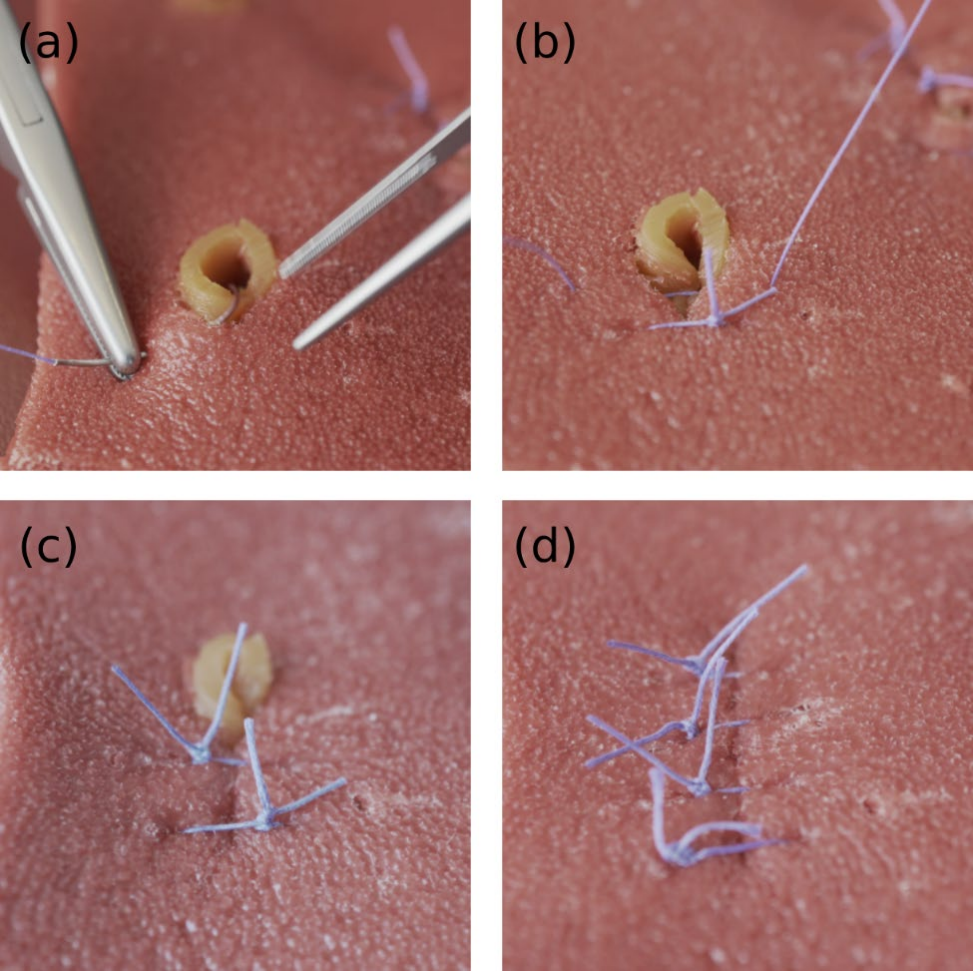
Different steps of the surgical procedure (a) penetration of the silicone “muscle” layer with the needle (b), (c) closing the perforation with simple interrupted stitches (d) closed perforations using 3–4 simple interrupted stitches. The muscularis layer is properly closed by sewing, while the mucosa layer remains untouched and is only pushed to the inside of the stomach

Silicone model A was tested in an open setting. Primary closure was done with interrupted stitches using Vicryl 3 − 0. The perforation could be easily closed with 3–4 simple interrupted stitches: the muscle layer III was sewed and the mucosa layer I was invaginated (Fig. 11).

After suturing the muscularis, the in vivo stomach wall shows an overlapping of the lining layer. This is necessary to close the perforation properly and could not be fully reproduced with the silicone model due to its lesser flexibility. The damage to the silicone layer by the thread (tear strength) was slightly higher than in porcine tissue.

A big advantage of the silicone model is that one can use it time-independent. No additional storage options (fridge) or hygienic requirements (laboratories) are necessary.

The model can be used several times/ repeatedly, depending on the number of holes produced and different suture techniques with different instruments and suture material can be practiced.

The two major layer (muscularis and mucosa) and their different treatment when it comes to closure of the perforation could be reproduced. Thereby, the moveable middle layer II is of help.

Silicone models of similar kind were shown to be used beneficially in surgical training with significant improvements in surgical skills [24, 25]. In particular, [25] showed that silicone models are especially suitable for training of advanced suturing techniques. Therefore, we expect the presented stomach perforation model to also have a positive impact on the surgeon’s performance.

## Discussion

This project aims at developing a silicone model of a gastric perforation that can be used to train sewing techniques in an open or laparoscopic surgery. Three different silicone models were constructed and characterised. The models consist of two respectively three layers of silicones and were mounted in a frame. They attempt to resemble the properties of a real gastric perforation while being simple. Within this study, subjective haptic tests as well as objective mechanical needle penetration tests were conducted to evaluate in which aspects the silicone models can mimic the real stomach wall.

### Silicone model fabrication

For the silicone model fabrication, only a small budget is required. The mold can be produced via a low budged 3D printer and the costs for support tools are marginal. The silicone is relatively affordable and an additional heat plate for silicone hardening is not necessary, inasmuch as the heat plate of the 3D printer can be used. Silicone models for sinus surgery [25] and renal tumor [24] were produced in a similar way and were shown do deliver a good model quality.

The different layer dimensions of the silicone model can be calculated very easily. The mixing of the silicone components is not challenging and the adhesion between the silicone layers is self-running, which is especially important for the submucosa (Fig. 2, model A, layer II). For the post-processing step, dexterity is needed to separate the protrusion from layer III. Altogether, the model manufacturing process is a compact forthcoming open source solution.

### Haptic test and needle penetration test

The results of the haptic evaluation favour silicone model A. Its overall appearance and behaviour during the sewing process are the closest to the cadaveric porcine stomach. Silicone model B was generally too stiff and when force was applied on the thread, the thread started to tear out and cut through the model.

The thin silicone layer in sample A (layer II) that simulates the submucosa layer is an additional feature that makes this model in particular realistic. It allows for a transverse elastic sliding between mucosa and muscularis externa. However, due to the missing wet environment, the suture material does not glide that easily through the tissue than through a real stomach wall. A potential improvement is the application of silicone oil on the sewing site to make the environment more slippery.

In contrast to the haptic tests, the results of the mechanical testing are more ambiguous. The loading part favours silicone model B as the most similar model to porcine stomach. Sample curve B shows a similar maximum force to the real stomach samples, corroborated by statistical analysis while not reproducing the initial toe region and needle retraction characteristics. In the unloading part, models A (statistically significant) and C (not statistically significant) were similar to the stomach samples. By exhibiting a smaller standard deviation, they are also more consistent in their mechanical properties.

To sum it up, the haptic evaluation clearly favours model A whereas the needle penetration tests brought up no distinct overall favourite. The conclusion from these results are drawn accordingly: For general surgical training where the model is touched, deformed and sewed, silicone model A should be used. In cases where a more precise penetration behaviour is needed, silicone model B should be used.

### Limitations

- This study was performed on porcine stomach as reference assuming its mechanical properties are close to human stomach. While [13] concluded, operation on the pig’s gastrointestinal system is similar to one on human, the comparison of mechanical properties between human and pig`s stomach has not been sufficiently done yet.
- The developed models cannot cover the changing structure within the stomach. As the different parts of the stomach have different functions, the thickness of the layers vary across the stomach surface. Additionally, the vascularisation and other smaller structures of the stomach were neglected as this would have meant a disproportional increase of complexity.
- All tests were performed in vitro. In vivo characteristics of the stomach wall may be different. However, [30] obtained similar reproducible results regarding mechanical properties of the human gastrointestinal tract for cadaveric and surgically removed stomach under certain storage conditions.
- Tests with the needle and thread were done with a specific needle and threat. The suture material may differ from hospital to hospital as well as from surgeon to surgeon and may influence the test results.
- As a reference, stomach from healthy pig was used. A stomach wall with a gastric perforation may have different characteristics due to inflammation processes. Those pathological conditions have not been reproduced in this silicone model.

## Conclusion

In conclusion, the newly developed silicone model A can be beneficially used for general training of gastric perforation surgery. It was shown that it has similar properties as a pig’s stomach and, thus, as a human stomach. The silicone model can be easily copied at low-costs.

The study shows that haptic considerations alongside with mechanical testing are beneficial methods to characterize and rank anatomical training models in order to design them as realistic as possible.

## Practice Points

Up to 5 short bullet points that summarise the key messages of the article should be included.

- New surgical training model
- Specific for training of gastric perforation sewing
- Model of low costs
- Easily reproducible and reusable
- Improvement in medical skills training

## Electronic supplementary material

Below is the link to the electronic supplementary material.

Supplementary Material 1

Supplementary Material 2

Supplementary Material 3

## Data Availability

The datasets generated during and/or analysed during the current study are available from the corresponding author on reasonable request.
