# Supplementary material for: A mechanically validated open-source silicone model for the training of gastric perforation sewing
Source: BMC Med Educ. 2023 Apr 19;23:261. doi: 10.1186/s12909-023-04174-8 (PMC10116820; doi:10.1186/s12909-023-04174-8)
Supplement: Supplementary file 1 — Supplementary Material 1 [file 12909_2023_4174_MOESM1_ESM.docx]

# Appendix

A photo gallery showing the production of a silicone model is shown in (supplementary figure 1).

Place supplementary figure 1 here.

A table shows specific numerical values of the maximal force related to the needle penetration test (supplementary table 1).
