# Supplementary material for: A mechanically validated open-source silicone model for the training of gastric perforation sewing
Source: BMC Med Educ. 2023 Apr 19;23:261. doi: 10.1186/s12909-023-04174-8 (PMC10116820; doi:10.1186/s12909-023-04174-8)
Supplement: Supplementary file 2 — Supplementary Material 2 [file 12909_2023_4174_MOESM2_ESM.docx]

# Supplementary figure


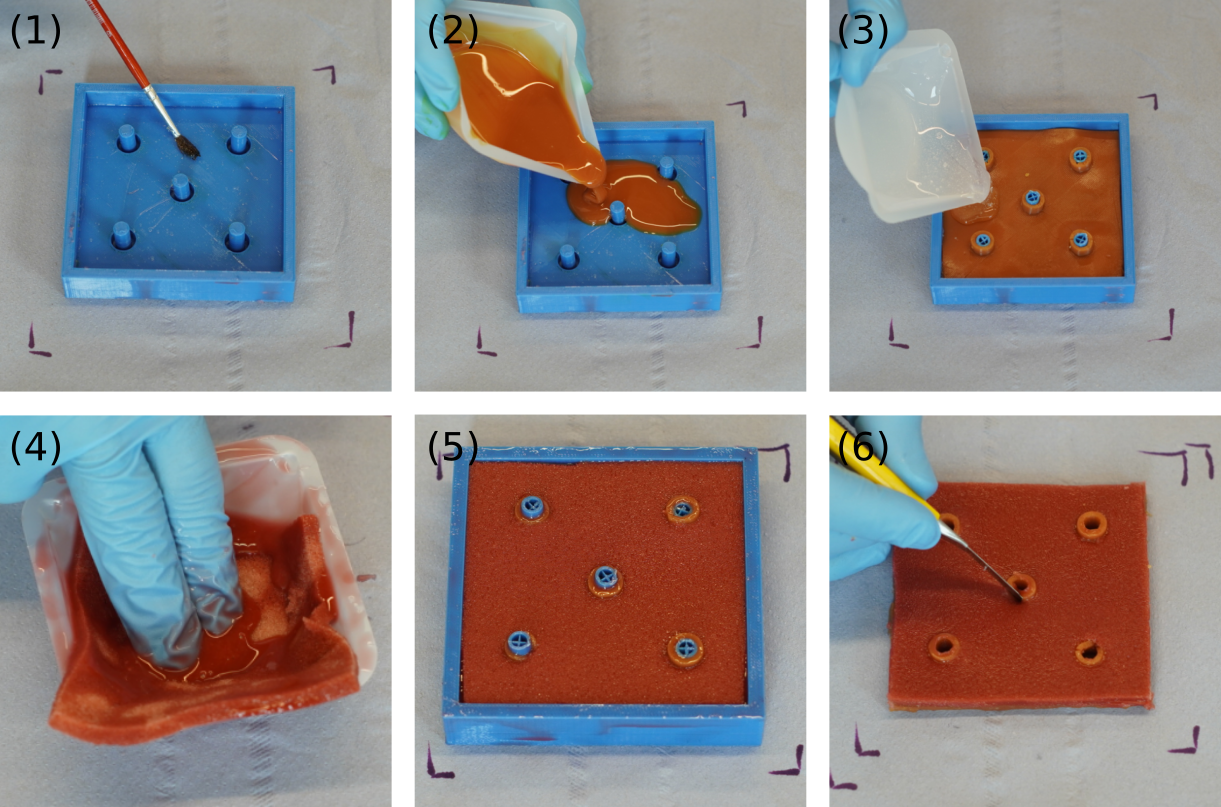


**Supplementary figure 1:** fabrication process: Step (1): Lubrication of the mold with oil. Step (2): Casting of layer I. Step (3): Flipping the first layer within the mold and casting of layer II (model A only). Step (4): Soaking the PU foam in silicone. Step (5): Introducing the soaked PU foam and casting layer III. Step (6): After curing, the model is removed from the mold. The protrusion were separated with a scalpel from the sidewalls of layer II and III.
