# Supplementary material for: A mechanically validated open-source silicone model for the training of gastric perforation sewing
Source: BMC Med Educ. 2023 Apr 19;23:261. doi: 10.1186/s12909-023-04174-8 (PMC10116820; doi:10.1186/s12909-023-04174-8)
Supplement: Supplementary file 3 — Supplementary Material 3 [file 12909_2023_4174_MOESM3_ESM.docx]

# Supplementary Tables

Supplementary Table 1: Overview of all numerical values of the maximum force in newton related to the needle penetration test.

| **N [N]** | **A [N]** | **B [N]** | **C [N]** |
| --- | --- | --- | --- |
| 1.768 | 0.561 | 3.291 | 1.154 |
| 3.65 | 0.705 | 1.907 | 1.03 |
| 2.85 | 0.585 | 1.365 | 0.82 |
| 2.919 | 1.098 | 2.772 | 0.668 |
| 2.498 | 1.029 | 2.009 | 0.862 |
| 1.889 |  |  |  |
| 2.266 |  |  |  |
| 1.902 |  |  |  |
| 2.37 |  |  |  |
| 2.711 |  |  |  |
| 1.912 |  |  |  |
| 2.641 |  |  |  |
| 2.775 |  |  |  |
| 2.857 |  |  |  |
| 2.227 |  |  |  |
| 2.207 |  |  |  |
| 2.185 |  |  |  |
| 1.983 |  |  |  |
| 2.06 |  |  |  |
| 2.204 |  |  |  |
| 1.403 |  |  |  |
| 1.887 |  |  |  |
| 2.346 |  |  |  |
| 2.403 |  |  |  |
| 1.51 |  |  |  |
